# The Effect of Leaf Stacking on Leaf Reflectance and Vegetation Indices Measured by Contact Probe during the Season

**DOI:** 10.3390/s17061202

**Published:** 2017-05-24

**Authors:** Eva Neuwirthová, Zuzana Lhotáková, Jana Albrechtová

**Affiliations:** 1 Department of Experimental Plant Biology, Faculty of Science, Charles University in Prague, Vinicna 5, 128 44 Prague 2, Czech Republic; eva.neuwirthova@natur.cuni.cz (E.N.); zuzana.lhotakova@natur.cuni.cz (Z.L.); 2 Institute of Botany, Academy of Sciences, Zámek 1, 252 43 Pruhonice, Czech Republic

**Keywords:** broadleaved trees, leaf optical properties, leaf traits, planar leaf, *Populus tremula*, VIS-NIR spectroscopy, spectroradiometer, spectral ranges, *Salix caprea*, seasonal dynamics, SWIR

## Abstract

The aims of the study were: (i) to compare leaf reflectance in visible (VIS) (400–700 nm), near-infrared (NIR) (740–1140 nm) and short-wave infrared (SWIR) (2000–2400 nm) spectral ranges measured monthly by a contact probe on a single leaf and a stack of five leaves (measurement setup (MS)) of two broadleaved tree species during the vegetative season; and (ii) to test if and how selected vegetation indices differ under these two MS. In VIS, the pigment-related spectral region, the effect of MS on reflectance was negligible. The major influence of MS on reflectance was detected in NIR (up to 25%), the structure-related spectral range; and weaker effect in SWIR, the water-related spectral range. Vegetation indices involving VIS wavelengths were independent of MS while indices combining wavelengths from both VIS and NIR were MS-affected throughout the season. The effect of leaf stacking contributed to weakening the correlation between the leaf chlorophyll content and selected vegetation indices due to a higher leaf mass per area of the leaf sample. The majority of MS-affected indices were better correlated with chlorophyll content in both species in comparison with MS-unaffected indices. Therefore, in terms of monitoring leaf chlorophyll content using the contact probe reflectance measurement, these MS-affected indices should be used with caution, as discussed in the paper. If the vegetation indices are used for assessment of plant physiological status in various times of the vegetative season, then it is essential to take into consideration their possible changes induced by the particular contact probe measurement setup regarding the leaf stacking.

## 1. Introduction

Leaf optical properties (reflectance, absorbance and transmittance) are defined as a ratio of incoming light, which is reflected, absorbed or transmitted by a leaf. Leaf optical properties, particularly reflectance, are exploited by remote sensing techniques to gain information about photosynthetic pigment contents, water content or other biochemical compounds in vegetation [1,2,3]. In laboratory conditions, optical properties at the leaf level are measured by a spectroradiometer usually equipped with an integrating sphere or with a contact probe. Although some spectroradiometers operate in different spectral ranges, the most common portable spectroradiometers for a laboratory and field measurement of optical properties (e.g., FieldSpec 4 and ASD) operate from visible (VIS) to infrared wavelengths of electromagnetic spectrum (350–2500 nm). A non-contact measurement with an optical sensor mounted in nadir above a sample has been used recently less frequently. Based on a measurement setup we gain information about reflectance and transmittance when using the integrating sphere [4,5,6,7,8] or only about reflectance, when using the contact probe [9,10]. Measurements with a contact probe have an advantage of fast processing comparing to time-consuming measurement with an integration sphere.

For laboratory measurement of leaf optical properties using a contact probe it is possible to use harvested planar leaves separately as individual, single leaves [9] or in a stack of several leaves [11]. Contact reflectance measurement in conifers can be measured on needles attached on twigs [12] or only needles cut off twigs [13]. Under the non-contact measurement setup with an optical sensor mounted less than one meter in nadir of the whole plant sample [9], usually harvested planar leaves in a stack [14] or whole seedlings are used [15]. Apart of the measurement on a single leaf in all mentioned setups, the leaves in the field of view of an optical sensor overlay at least partly. The leaf overlapping introduces additional variability in leaf spectra due to higher LAI (leaf area index) [16] of the sample and accumulation of its mass per an area unit. Furthermore, we suppose a different mode of scattering inside a single leaf in comparison to the mode of scattering at the interface of individual leaves in a stack what may influence the resulting sample reflectance. Spectral data acquired at a leaf level by a spectroradiometer then serve as an input in radiative transfer (RT) models at a leaf level (e.g., PROSPECT [17]) and can be coupled with RT models at a canopy level (e.g., PROSAIL [18] or DART [19]) and provide better understanding of airborne remote sensing data in large-scale studies [20]. From this point of view, the effect of leaf stacking and overlapping on its reflectance is important if the spectra from the leaf level would be scaled up to the canopy level.

Vegetation indices are used for assessment of plant physiological status. Indices are derived from reflectance in absorption maxima of particular biochemical leaf compounds [5,9,14] and correlate with the chlorophyll content [4,5,21,22], carotenoid and anthocyanin contents [23] or a ratio of carotenoids to chlorophyll [24] and water content [9]. The most frequently used vegetation indices are calculated as: (1) simple ratios of reflectance in individual wavelengths (in nm), Rλ (reflectance in λ wavelengths), e.g., BGI (Blue green pigment index) = R450/R550, G (Greenness index) = R554/R677 [25]; or (2) normalized differences of reflectances in individual wavelengths, e.g., PSNDc (Pigment specific normalized difference) = (R800 − R470)/(R800 + R470) [24]. Simple ratio pigment-related indices are usually computed from wavelengths from VIS region where the photosynthetically active radiation is absorbed [26,27]. Therefore, these indices are sensitive to changes in photosynthetic pigments, particularly in the chlorophyll content [28]. For example, absorption properties of chlorophyll in 700 nm and 550 nm [23] were included in CRI 550, or CRI 700 (Carotenoid concentration indices) [22], which are commonly used for chlorophyll content estimates.

Normalized difference indices for chlorophyll estimate include red edge wavelengths (680–750 nm), where the reflectance sharply increases [29], and wavelengths from near infrared (NIR) region (740–1140 nm) [10]. Particularly, reflectance in NIR is insensitive to changes in the chlorophyll content, however, sensitive to internal leaf structure and water content [6]. Thus, indices including wavelengths from NIR enable an efficient pigment content estimate, e.g., OSAVI [30], Gitelson2, Datt [31], PSSRa, and PSNDb [24], while these indices do not take into account changes in leaf anatomical properties. On the other hand, accumulation of leaf structural compounds contributes to reflectance changes in infrared wavelengths (NIR, SWIR). In spectroscopic studies the leaf content of dry matter and structural compounds is usually expressed as leaf mass per area (LMA) [32]. Vegetation indices can also serve for empirical LMA retrieval [33]. However, the physical approaches are more common, provided that LMA is one of the main parameters for leaf level radiative transfer models, such as PROSPECT [17]. Reflectance in particular regions in NIR is further influenced by leaf water status [9] that is frequently expressed as an equivalent of water thickness (EWT (water content per unit of leaf area), e.g., [34]). The leaf reflectance in NIR also decreases with leaf senescence [4] and depends on the leaf orientation (adaxial or abaxial side of a leaf) towards a sensor [35]. Regarding the leaf orientation, VIS is more effectively absorbed if incoming from the adaxial leaf side than from the abaxial one. The reason is that the chlorophyll is unevenly distributed within a leaf with a dorsiventral structure when more chlorophyll is below the adaxial epidermis in the dense palisade parenchyma [35]. On the other hand, the situation in NIR is different—more radiation is transmitted by the leaf and less is reflected when the leaf is illuminated from the abaxial side than from the adaxial one. This effect is probably caused by the different density of mesophyll cells and intercellular spaces in palisade and spongy parenchyma of a dorsiventral leaf, when dense palisade parenchyma is adjacent to the adaxial side while a loose spongy parenchyma is facing to the abaxial side.

Leaf optical properties change during the season depending on leaf phenology, accumulation of pigments, development of anatomical structure and leaf senescence at the end of the season. However, leaf optical properties and the shape of the spectral curve can change in a different manner in particular spectral ranges—VIS, NIR and SWIR [10] and thus, they can affect differently absolute values of vegetation indices. The reflectances in VIS and NIR decreased up to 5% and 30% as it was shown for *Vitis vinifera* L. [4] and *Picea abies* L. Karst [13], respectively, depending on the leaf/needle senescence. While in SWIR, the reflectance increased up to 20% in *Populus* ssp. [9] and was connected with water deficit [26]. The seasonal difference in the reflectance curve in VIS range influences the values of vegetation indices, which correlate with physiological parameters (e.g., photochemical reflectance index PRI = (R570 − R530)/(R570 + R530) correlating with the light use efficiency) [36]. For example, the normalized difference vegetation index (NDVI) in *Quercus rubra* L. and *Betula papyrifera* Marsh. [37] and PRI in selected tropical evergreen species [36] reached their maximum values in July and then values decreased at the end of the season. The strength of the relationship between vegetation indices and the real chlorophyll content may vary during the season and could depend on a leaf type, e.g., planar or needle-like leaves [5]. The relationship between PRI and light use efficiency in *Pinus strobus* L. decoupled in early spring due to chlorophyll and carotenoids recovery after winter and then it followed a good fit for a linear function [38]. However, there has not been done yet an extensive study comparing numerous vegetation indices based on leaf reflectance acquired by a contact probe during a vegetation season.

Measuring leaf reflectance in a different setup (a single leaf or more leaves in a stack) usually affects the reflectance curve as we can find several records throughout the literature (e.g., [11,16,26,39]). In the study of Blackburn [16], the leaf stacking was used in purpose to increase the range of LAI and chlorophyll content. Since the majority of studies including contact probe measurements are using only one measurement setup (either a single leaf or a leaf stack) we focused on comparison of both setups on two temperate broadleaved tree species with a similar dorsiventral leaf structure growing in the same field conditions. Moreover, there are several gaps in knowledge on dynamics of different spectral indices during the vegetation season when measuring the leaf reflectance with the contact probe. Considering that a reflectance in particular spectral ranges (VIS, NIR and SWIR) changes with a different extent during the vegetation season, we assume that this effect on values of spectral indices is not negligible. Therefore, filling this gap in knowledge will contribute to interpretation of spectral data acquired by the contact probe at a leaf level in purpose to assess temporal changes in the physiological status of vegetation or leaf phenology.

Our aim was to compare the leaf spectral reflectance measured by the contact probe on a single leaf and a stack of five leaves of two broadleaved tree species in VIS, NIR and SWIR spectral ranges during the vegetative season and to test if and how selected vegetation indices differ under these two measurement setups. We asked following research questions:

(1) How does the measurement setup (a single leaf or a leaf stack) influence a reflectance curve in selected spectral ranges (VIS, NIR and SWIR)?
(2) Does the difference between the reflectances measured on a single leaf and a leaf stack in the above selected spectral ranges differ during the season?
(3) Are the water- and pigment-related indices affected by the contact probe measurement setup (a single leaf or a leaf stack) during the season?

## 2. Materials and Methods

### 2.1. Study Site

The study was conducted at non-reclaimed post mining sites in the Sokolov coal mining district (50°14′21″ N, 12°39′24″ E) in the western part of the Czech Republic, Central Europe. The study area was located at an altitude of 500–600 m a.s.l., with mean annual precipitation of 650 mm and mean annual temperature of 6.8 °C [40]. The study site was covered by spontaneous woody vegetation dominated by European aspen (*Populus tremula* L.) and goat willow (*Salix caprea* L.). Due to the higher trichome density on the abaxial leaf side and lower specific leaf area, *S. caprea* leaves have a better xeromorphic adaptation than *P. tremula* leaves. However, both leaves exhibit a similar dorsiventral structure, i.e., mesophyll is differentiated into palisade and spongy parenchyma. These two species of broadleaved deciduous trees were sampled during the vegetative season from May to October 2014; particular sampling dates were: 26 May, 17 June, 8 July, 12 August, 9 September and 1 October. The trees were approximately 20–25 years old, and 5–7 m tall. Fully developed leaves were taken from the crown transition zone between sun-exposed and shaded parts of a tree crown. Fresh leaves from 10 trees of each species were clipped using telescopic pruning poles, put into the zip lock plastic bags, cooled and immediately transported to the laboratory. The spectra were measured within 24 h after the collecting. The leaf samples for assessment of chlorophyll and water contents were put into plastic vials, cooled and further processed as described below.

### 2.2. Leaf Spectra Measurements

Spectral measurements were conducted using an ASD FieldSpec 4 Wide-Res portable spectroradiometer (ASD Inc., Boulder, CO, USA). Each spectral measurement was preceded by a dark current measurement and a white reference measurement using a 99% Spectralon panel. The radiance spectra of the leaf reflectance between 350 and 2500 nm were normalized against white reference to produce relative reflectance spectra.

The leaves were placed on a spectrally black surface to minimize the background spectral noise or radiation transmitted through them. A fiber optic contact probe (ASD Plant Probe; ASD Inc., USA) was placed on a leaf surface, which was only illuminated by a constant light source inside the contact probe. The scan average on the spectroradiometer was set to 15 to avoid overheating the scanned foliage [9]. Five leaves per tree were used for spectral analyses. First, spectral reflectance of each of those five leaves was measured separately. Then, the five leaves were arranged into a stack by their adaxial surfaces facing to the top and the reflectance was measured again. Five independent measurements were taken on different parts of each sample. For each leaf or a leaf stack a median reflectance value was calculated and then the curve was used for further data analysis.

### 2.3. Assessment of Biophysical Leaf Traits

After reflectance measurements, fresh leaves were scanned and the leaf area determined by ImageJ freeware. Then the leaves were dried and weighed for assessment of LMA (leaf mass per area unit). The leaf water content was determined as the percentage of water in the fresh leaves (the fraction of weight decrease after drying at 80 °C for 48 h until constant weight) and EWT (equivalent water thickness) was calculated according to [41]. The total chlorophyll content was determined in dimethylformamide extract using a spectrophotometric assessment based on the equations reported in [42].

### 2.4. Spectral Ranges and Vegetation Indices

According to Cavender-Bares [10] we divided full leaf spectra to four spectral ranges: VIS (400–700 nm), NIR (740–1140 nm), SWIR1 (1500–1800 nm) and SWIR2 (2000–2400 nm). In purpose of quantification the effect of the MS in those spectral ranges, the difference between the reflectances measured on a leaf stack of 5 leaves and a single leaf (i.e., 1 leaf), (R5L subtraction R1L = ∆R_5L–1L_), was calculated for each wavelength. Thus, the value of ∆R_5L–1L_ expresses the additional variability in leaf spectra introduced by increasing LAI, LMA and the internal leaf surface area as a potential scattering interface. Stacking of the leaves in a laboratory can be regarded as approximation of the contact measuring setup to the measurement at the canopy level.

Vegetation indices were computed in the statistic program R-3.2.3 (the open source version of Statistica) from the reflectance measured in both measurement setups (a single leaf, a leaf stack). In total, 74 pigment-related indices and 7 water-related indices were determined. For all tested indices and their references see Table 1. For demonstration of temporal dynamics of the selected indices, their values were transformed into standardized z-scores to enable their comparability and independence of their physical dimensions. Z-scores generally express how far from the mean the particular value is in terms of the standard deviations.

### 2.5. Statistical Analyses

All statistical analyses were performed with NCSS 9 software (NCSS, LCC, Kaysville, UT, USA). Descriptive statistics for the reflectance of a single leaf and a stack of leaves were calculated separately for VIS, NIR, SWIR1 and SWIR2 spectral ranges. The effect of the sampling date on the value of difference ∆R_5L–1L_ and vegetation indices was tested by one-way ANOVA and Tukey–Kramer (in case of a normal distribution) or Kruskal–Wallis (if not normal distribution) tests. All differences were classified as significant if *p* < 0.05.

## 3. Results and Discussion

**Figure 1:**
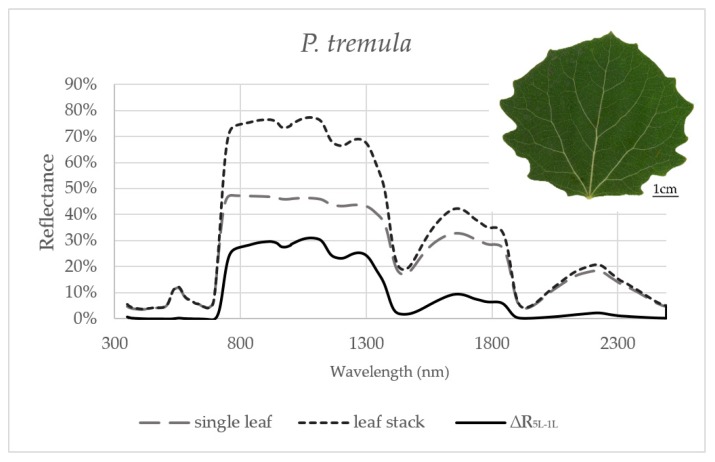
Averaged reflectance curves measured by a contact probe for a single leaf of *P. tremula*, a leaf stack and a difference (∆R_5L–1L_) between the reflectance measured on a leaf stack (5L) and a single leaf (1L). The mean reflectance (%) during the six months in *P. tremula* (*n* = 10 trees).

**Figure 2:**
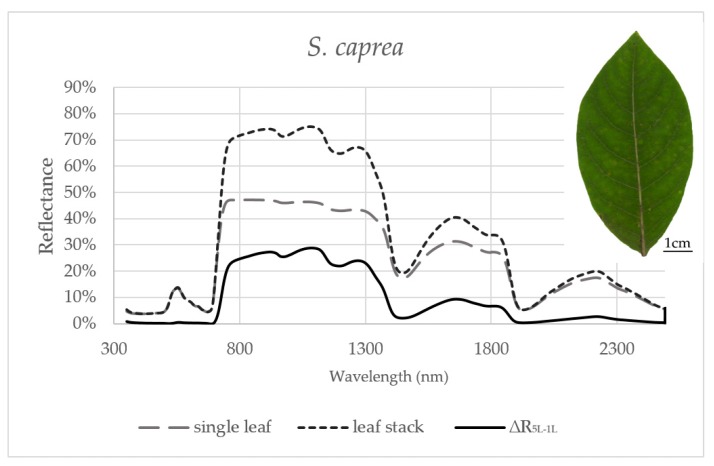
Averaged reflectance curves measured by a contact probe for a single leaf of *S. caprea*, a leaf stack and a difference (∆R_5L–1L_) between the reflectance measured on a leaf stack (5L) and a single leaf (1L). The mean reflectance (%) during the six months in *S. caprea* (*n* = 10 trees).

**Figure 3:**
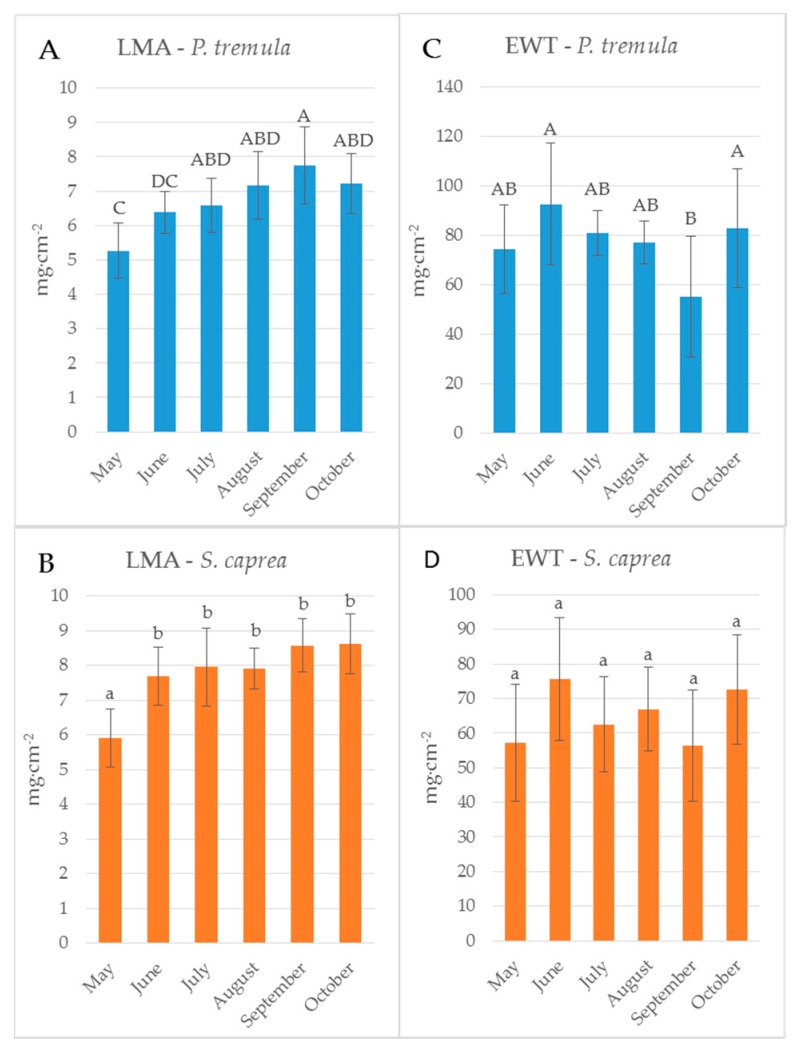
Seasonal dynamics of leaf traits of: *P. tremula* (**A**,**C**); and *S. caprea* (**B**,**D**). LMA, leaf mass per area (**A**,**B**); and EWT, equivalent water thickness (**C**,**D**). Different letters above the columns correspond to a significant difference, One-way ANOVA, Tukey–Kramer test. Significant differences if *p* < 0.05.

**Figure 4:**
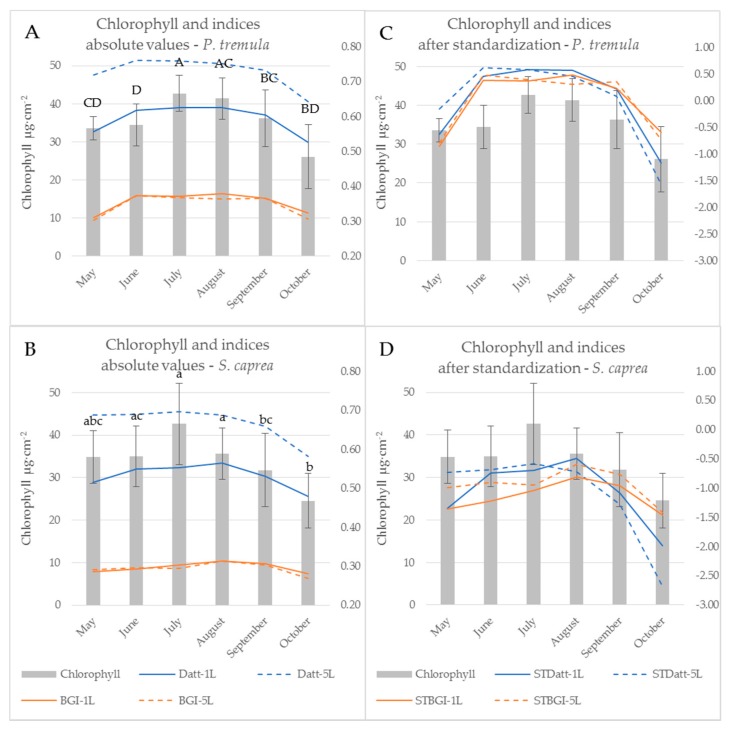
Chlorophyll a + b content of *P. tremula* (**A**,**C**) and *S. caprea* (**B**,**D**) during the vegetative season (bars, left axis) and a seasonal course of selected chlorophyll indices (lines; right axis). Datt is presented as an index affected by the measurement setup, BGI as an index unaffected by the measurement setup. Left graphs (**A**,**B**) show absolute values of indices computed from the contact probe reflectance of a single leaf (1L) and a leaf stack (5L). Right graphs (**C**,**D**) show identical indices after z-score standardization. Chlorophyll content: different letters above the columns correspond to a significant difference, One-way ANOVA, Tukey–Kramer test. Significant differences if *p* < 0.05.

Reflectance curves acquired by a contact probe measurement on a single leaf and a leaf stack and the difference between them (∆R_5L–1L_) averaged from all measurements during the season are presented for *P. tremula* and *S. caprea* in Figure 1 and Figure 2, respectively. The results are very close to each other for both species, which is in accordance with the following facts: (1) The leaves of both tree species had a similar anatomical structure of a typically dorsiventral leaf, which also confirmed their similar values of LMA (Figure 3A,B); (2) Both tree species grew in close vicinity at one site, thus having the same environmental conditions. This is in accordance with the seasonal pattern of the EWT (Figure 3C,D), which was the same for both species; depending probably on precipitation; (3) Both species share the life strategy of pioneer species and similar phenology that is obvious from the chlorophyll content and LMA seasonal dynamics (Figure 3A,B and Figure 4A,B).

### 3.1. Effect of the Measurement Setup on the Reflectance Curve in Selected Spectral Ranges

**Figure 5:**
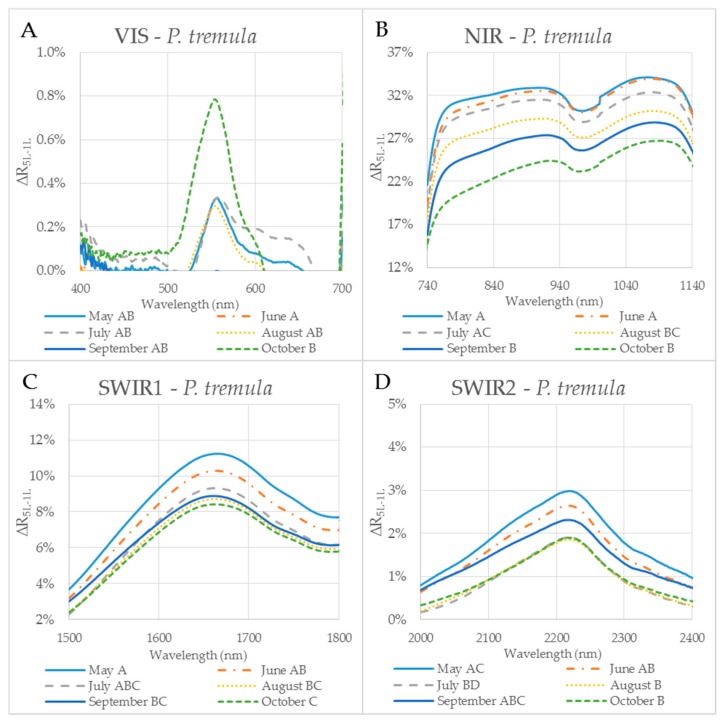
The difference (∆R_5L–1L_) between the reflectances measured on a leaf stack (5L) and a single leaf (1L) during the vegetative season, represented by curves for 6 months (May–October) for selected spectral ranges in *P. tremula*. The mean value of ∆R_5L–1L_ for each month from May to October within: (**A**) VIS (400–700 nm); (**B**) NIR (740–1140 nm); (**C**) SWIR1 (1500–1800 nm); and (**D**) SWIR2 (2000–2400 nm). Different capital letters by months´ names correspond to a significant difference in ∆R_5L–1L_, computed from the mean reflectance values in the particular spectral range and the month of sampling. One-way ANOVA, Tukey–Kramer test. Significant differences if *p* < 0.05.

**Figure 6:**
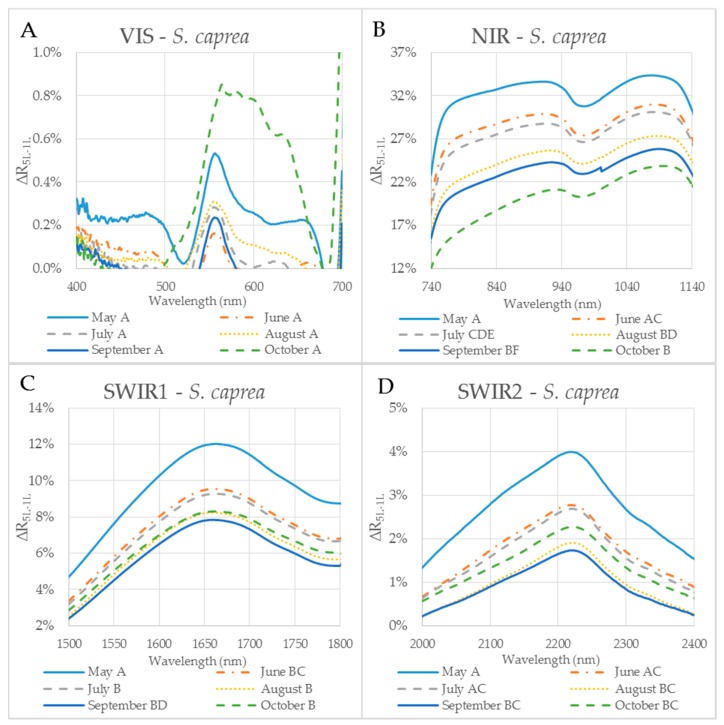
The difference (∆R_5L–1L_) between the reflectances measured on a leaf stack (5L) and a single leaf (1L) during the vegetative season, represented by curves for 6 months (May–October) for selected spectral ranges in *S. caprea*. The mean value ∆R_5L–1L_ for each month from May to October within: (**A**) VIS (400–700 nm), (**B**) NIR (740–1140 nm) and (**C**) SWIR1 (1500–1800 nm) and (**D**) SWIR2 (2000–2400 nm). Different capital letters by months´ names correspond to a significant difference in ∆R_5L–1L_, computed from the mean reflectance values in the particular spectral range and the month of sampling. One-way ANOVA, Tukey–Kramer test. Significant differences if *p* < 0.05.

In order to capture the effect of the MS on reflectance curves, we designed for the purpose of the present study a metric corresponding to the difference between the reflectances measured on a leaf stack of 5 leaves and a single leaf, i.e., reflectance difference (∆R_5L–1L_). The highest mean value of the reflectance difference ∆R_5L–1L_ for both species was up to 30% in NIR (Figure 5B and Figure 6B) similarly as in the study by Blackburn [16]. We suppose that a higher ∆R_5L–1L_ was caused by an increased content of structure components in stacks of leaves (e.g., increase in LAI and/or LMA) as reported in other studies [26,82,83]. Relatively small reflectance difference ∆R_5L–1L_ in SWIR 1 (up to 10%) and SWIR2 (up to 5%) (Figure 5C,D and Figure 6C,D) could be explained by water-related absorption depending on EWT [34], which increases with each additional leaf layer [11]. The reflectance in VIS was up to 15% in both measurement setups (Figure 5A and Figure 6A). A similar trend was observed in a stack of 12 leaves in the study by [39]. The value of ∆R_5L–1L_ in VIS was close to zero what confirmed that structure and function of a planar leaf is very well adapted to absorb light in the VIS range [84], regardless if the light comes directly down on a leaf or is transmitted from an upper leaf layer. The radiation reflected from the adaxial leaf side from leaves in a stack is again reflected from the spongy parenchyma of upper leaves, which is helpful for achievement of maximum utilization of light in a canopy [85].

The descriptive statistics for the reflectance difference ∆∆R_5L-1L_ in selected spectral ranges (VIS, NIR, SWIR1 and SWIR 2), throughout the growing season from all samples, are presented in Table 2. Both *P. tremula* and *S. caprea* kept the similar values of ∆R_5L–1L_ in the identical spectral ranges what can be explained by the same growing conditions and living strategies of both tree species. The similarity between optical properties of *P. tremula* and *S. caprea* is also based on the field-observed biophysical leaf traits—total chlorophyll content, leaf mass per area and equivalent water thickness (Figure 3 and Figure 4), which were almost identical in both species. Moreover, the temporal trends of all studied leaf traits followed the same pattern for both species.

For both tree species, the maximum and the minimum values of ∆R_5L–1L_ were detected in NIR and VIS, respectively. The variance of ∆R_5L–1L_ in NIR was up to two orders higher than in VIS. Measuring the leaf reflectance under a different measurement setup (a single leaf or more leaves in a stack) usually affects the reflectance curve [26]. The reflectance of a leaf stack in NIR was higher about 20–30% than the reflectance of a single leaf as it was shown in the study by [11] when the stack was composed of four or six planar leaves (species not indicated). We confirmed the same effect of the measurement setup in our study using one and five leaves of *P. tremula* and *S. caprea*.

### 3.2. Seasonal Dynamics of the Difference between Reflectances of a Leaf Stack and a Single Leaf

The reflectance difference ∆R_5L–1L_ during the season in four selected spectral ranges (VIS, NIR, SWIR1 and SWIR2), is displayed in Figure 5A–D and Figure 6A–D for *P. tremula* and *S. caprea,* respectively. Differences in values of ∆R_5L–1L_ among measurements conducted in different months during the vegetative season were significant, but not in all cases. Although values of ∆R_5L–1L_ in VIS were lower than 1% in both species, the highest difference between values of ∆R_5L–1L_ was detected between 500 and 600 nm in October for both species (Figure 5A and Figure 6A). We suppose that it was most likely due to the progress of the leaf senescence accompanied by the chlorophyll decay shown for both species (Figure 4). In both species, the effect of the measurement setup was more pronounced at the end of the season, comparing to the beginning of the season, showing the highest values of ∆R_5L–1L_ in VIS in October. The ∆R_5L–1L_ values in NIR were considerably higher than the values in other spectral ranges (VIS, SWIR1 and SWIR2) during the whole season. This can be explained that the reflectance in NIR depends on sample structural parameters (LMA, LAI) and leaf sample thickness, which mainly differed between the two used measurement setups, a single leaf and a stack of leaves. During the season, ∆R_5L–1L_ in NIR declined in both *P. tremula* (Figure 5B) and *S. caprea* (Figure 6B). On one hand, we explain the decrease of ∆R_5L–1L_ value by declining NIR reflectance in both a single leaf and a stack of leaves (data not shown) in the consequence of leaf aging, similarly as was observed by [4] in *V. vinifera* and by [8] in *Alnus incana* L. Moench and *Betula pendula* L. On the other hand, we expected that the accumulation of the cell wall structural compounds documented by the trend of LMA increase (Figure 3A,B) would lead to a higher NIR reflectance. However, we can attribute the decrease of both, the single leaf reflectance and also a value of ∆R_5L–1L_ during the season, not only to absorption of NIR radiation by the structural compound themselves, but also to changing internal mesophyll structure (e.g., cell wall thickening). Modulating the size of the internal leaf surface may lead to a lower light scattering inside the leaf and, thus, a lower NIR reflectance.

The decrease of ∆R_5L–1L_ value in SWIR1 (Figure 5C and Figure 6C) and SWIR2 (Figure 5D and Figure 6D) kept the same pattern during the season for both species. From May to July the decrease in ∆R_5L–1L_ was gradual, however, from August to October the change in ∆R_5L–1L_ was insignificant and rather inconsistent. The seasonal changes in ∆R_5L–1L_ in SWIR could be partly explained by accumulation of structural compounds (LMA) similarly as in NIR, however, the effect was weaker. We attribute the fluctuation of value of ∆R_5L–1L_ in SWIR between August and October to irregular changes of EWT in both species (Figure 3C,D).

### 3.3. The Effect of the Measurement Setup on Vegetation Indices during the Season

**Figure 7:**
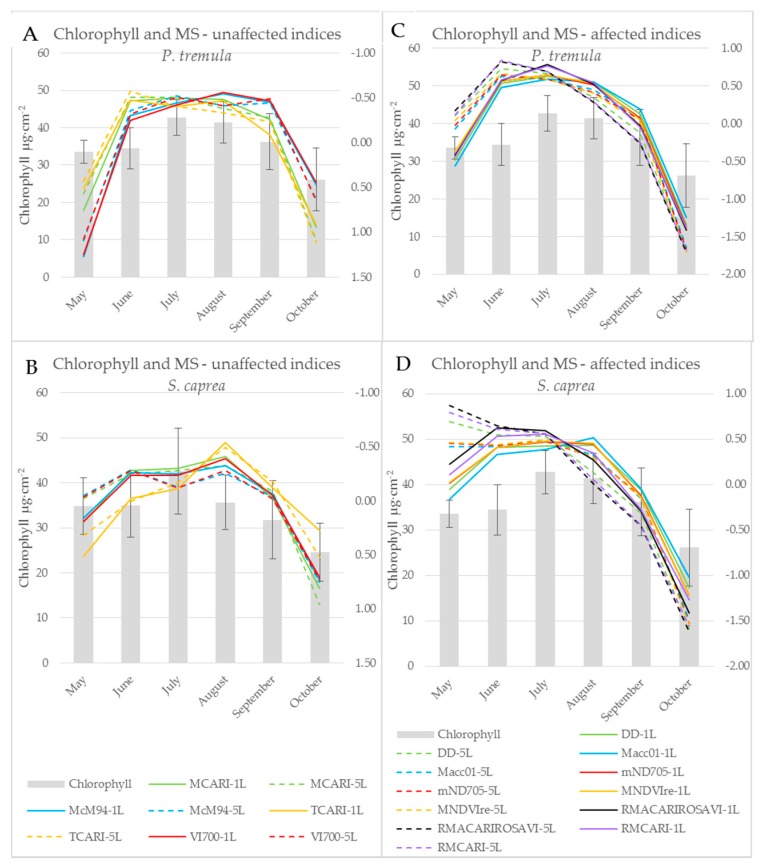
Chlorophyll a + b content of *P. tremula* (**A**,**C**) and *S. caprea* (**B**,**D**) during the season (bars; left axis) and a seasonal course of selected chlorophyll indices (lines; right axis) after z-score standardization. Left graphs (**A**,**B**) present indices unaffected by the measurement setup. Right graphs (**C**,**D**) present indices affected by the measurement setup. Graphs show absolute values after z-score standardization of indices computed from contact probe reflectance of a single leaf (1L-full lines) and a leaf stack (5L-dashed lines).

Subsequent paragraphs discuss differences between the water- and pigment-related indices computed from the reflectance acquired by both contact probe measurement setups (a single leaf, a leaf stack) during the vegetative season. Significant differences between indices calculated from both measurement setups during the season are expressed in Table 1. When the difference between vegetation indices obtained from two contact probe measurement setups was calculated, eight indices (BGI, CRI 550, CRI 700, MCARI, McM94, PRIm1, TCARI, and VI_[700]_) out of 85 indices tested were not affected by the measurement setup throughout the growing season in both tree species. All eight of these indices were derived from the reflectance in wavelengths up to 700 nm, i.e., in VIS. Five (BGI, MCARI, McM94, TCARI and VI_[700]_) out of those vegetation indices classified as unaffected by the measurement setup were chlorophyll-related and followed the seasonal course of chlorophyll leaf content in both species (see example of BGI in Figure 4 and Figure 7). The temporal variation of vegetation indices corresponded better with the leaf chlorophyll content in *P. tremula* in comparison to *S. caprea*. This difference between species is also documented by higher R^2^ values for linear regression between chlorophyll content and indices (Table 1) for *P. tremula*. In these five MS-unaffected chlorophyll indices, the R^2^ values for linear regression with the chlorophyll content were always higher for indices computed from a reflectance of a single leaf (Table 1).

On the other hand, indices including wavelengths from both VIS and NIR spectral regions (e.g., DD, Macc01, mND705, MNDVIRe, RMCARI, and RMACARIROSAVI) always showed a significant dependence on the measurement setup (a single leaf, a leaf stack). These indices also correlated significantly with the chlorophyll content (Table 1) and exhibited the good fit to a chlorophyll seasonal dynamics (see example of Datt in Figure 4). Here, the effect of the contact probe measurement setup was obvious (Figure 4A,B): stacking the five leaves significantly shifted the absolute values of the index comparing to measurement on a single leaf. We assume that the fact, if a particular index is affected by the contact probe measurement setup, is determined by the extent of contribution of lower leaves in the stack to resulting sample reflectance. Regarding this, Blackburn [16] showed that the lower leaves in the stack have a minor or negligible effect on overall reflectance, particularly in VIS. Therefore, the indices unaffected by the contact probe measurement setup were rather determined by the chlorophyll content in the uppermost leaf.

The z-score standardization of vegetation indices enabled to compare their seasonal course with the chlorophyll content dynamics irrespective of their different absolute values (Figure 7). However, we could still recognize different behaviour of MS-unaffected and MS-affected indices. The effect of the contact probe measurement setup on the latter ones was detectable particularly in the beginning of the season (May and June) and decreased towards the end of the season (Figure 7C,D and the *p*-values slightly increased since August, Table 1). In the case of processing vegetation indices and spectral data acquired under different contact probe measurement setups (regarding leaf stacking), we recommend to take into account this dependence on measurement setup particularly in the beginning of the season, even if the data standardization was applied.

The results presented above imply that vegetation indices, which involve wavelengths from VIS only, were almost unaffected by a contact probe measurement setup of a single leaf or a leaf stack, in comparison to indices operating with reflectance wavelengths from more than one spectral range. The dependence of vegetation indices on measurement setup is given by a larger, more contrasting difference in ∆R_5L–1L_ between spectral ranges used for index calculation (Figure 1). Vegetation indices including wavelengths from two spectral ranges with different value of ∆R_5L–1L_ were more dependent on a measurement setup in the beginning of the vegetation season than at the end of it (Figure 7C,D). The dynamic change of ∆R_5L–1L_ during the season may affect the correlation strength of these vegetation indices to leaf biophysical traits used for the estimation of a tree physiological status if the reflectance is measured at a leaf level. However, many of MS-affected indices, such as those presented in (Figure 7C,D) better correlated with chlorophyll content in both species in comparison with MS-unaffected indices (Table 1). Therefore, in terms of monitoring leaf biophysical traits using the spectroscopic approach, NIR reflectance should not be excluded and these MS-affected indices should be used with the above described caution.

Currently, there are emerging studies on spectra modeling or reverse radiative transfer modeling that combine leaf optical properties and biophysical traits from independent experiments or open access spectral libraries: LeuvenV and LOPEX datasets [86]; ANGERS, LOPEX and PANAMA datasets [87]; or various independent datasets (see Table 1 in [88]). These studies also test and implement vegetation indices derived from spectra provided in these databases [87]. We are convinced that it is always important to use the data acquired by the most comparable measurement setups. Concerning this, in our previous study of Potůčková [89], we emphasized that in case of processing spectra acquired by two different devices, a linear model transforming spectra between those devices is necessary. On the example of several vegetation indices we have demonstrated, that in most cases the measurement setup should be taken into account, even if the identical instruments (the spectroradiometer and the contact probe) and data standardization are used.

### 3.4. Leaf Stack as an Intermediate Step towards the Canopy Reflectance

Measuring reflectance on more leaves together in a stack in a present study had a similar effect on the leaf reflectance as changing a structural parameter N in the radiative transfer model PROSPECT [17]. Simulation of the leaf reflectance in PROSPECT includes structural parameter N, corresponding to a number of elementary homogeneous layers in a leaf [87]. Increasing of N parameter results in the increase of the reflectance values systematically in all spectral ranges [17]. Nevertheless, the effect of changes in leaf structural parameters on quantitative values of the reflectance in the VIS range was negligible in comparison to changes induced by pigment contents [83].

In some cases, we can consider the reflectance of a leaf stack as an intermediate step towards the canopy reflectance. Regarding that the change between the reflectance curve of a single leaf and a stack of leaves in our study across all studied spectral ranges (Figure 1 and Figure 2) was very similar to the effect of the increasing LAI in the canopy reflectance simulated by the radiative transfer model PROSAIL [90]. Although we suggest that stacking the leaves can partly approximate the contact probe reflectance measurement to the canopy level, we are aware that the reflectance derived from a leaf stack is not equal to the canopy-scale reflectance. By piling several layers of leaves we technically increased chlorophyll content, leaf area index and leaf mass per area unit (LAI and LMA). In addition, the effect of the internal leaf structure such as both volume and surface of intercellular spaces is enhanced in a leaf stack. These effects of a leaf stacking altogether can influence the correlation between leaf biophysical traits and leaf optical properties including vegetation indices, which we demonstrated with systematically lower R^2^ values for vegetation indices acquired from the reflectance of the leaf stack (Table 1). Moreover, it is important to mention that the contribution of leaf traits to the resulting leaf stack reflectance may depend on a leaf position within the stack [16]. On the other hand, the real canopy-scale reflectance is affected by additional factors, such as leaf clumping, leaf angle distribution, presence of non-photosynthetic structures (branches and twigs) and soil/understory reflectance background as summarized, e.g., by Homolová et al. [32]. In case of airborne remote sensing, the effect of atmosphere should be considered and atmospheric corrections should be made [91]. Currently, the appropriate upscaling of the reflectance from the single leaf level to the canopy level is usually performed by the coupling of radiative transfer models at the leaf (e.g., PROSPECT) and canopy levels (e.g., SAIL [92] or DART [19]).

## 5. Conclusions

The major influence of the contact probe measurement setup (one leaf or a stack of five leaves) on a leaf spectral curve was detected in NIR, the structure-related spectral range and a weaker effect was detected in SWIR1 and SWIR2, water-related spectral ranges. In VIS, the pigment-related spectral region, the effect of measurement setup was almost absent. Our results confirmed that in NIR, the reflectance of a leaf stack is about 25% higher than the reflectance of a single leaf. In a consequence of good adaptation of a planar leaf to absorb VIS radiation incident to its adaxial side (including the light transmitted from upper leaf layers), there was no influence connected with measurement setup in VIS. We concluded that the main effect on difference between the two measurement setups in NIR was induced by the change in sample structural parameters originating from additional leaves in the stack. On the contrary, in VIS, the chlorophyll content of leaves placed lower in the stack contributed less to the reflectance of the sample not affecting it significantly.

The considerable influence of a measurement setup was detected at the beginning of the season, according to the highest values of ∆R_5L–1L_ in May in all selected spectral ranges except VIS. The maximum values of ∆R_5L–1L_ in VIS observed in October were most likely due to the leaf senescence and chlorophyll content decrease in both species. The values of ∆R_5L–1L_ in NIR and SWIR were higher at the beginning of the season and diminished towards the end of the season. We attribute the decrease of ∆R_5L–1L_ values during the season to the accumulation of the cell wall structural compounds documented by the trend of LMA increase. We suppose that the cell wall thickening modulates the size of the internal leaf surface and may lead to lower scattering inside the leaf and, thus, lowering the NIR reflectance. We assume that the seasonal changes in values of ∆R_5L–1L_ in SWIR could be partly explained by the fluctuation of leaf equivalent water thickness and the accumulation of structural compounds, similar to in NIR, however, the effect was weaker.

The strong effect of stacking up the leaves while measuring reflectance by a contact probe should be regarded in studies where the reflectance is measured on a stack of leaves. We clearly showed that vegetation indices involving wavelengths up to 700 nm were independent of the measurement setup during the vegetation season. On the contrary, indices combining wavelengths from both VIS and NIR were affected by the measurement setup throughout the season. We assume that the sensitivity of a particular index to the contact probe measurement setup is given by the contribution of lower leaves in the stack to resulting sample reflectance. This effect of leaf stacking contributed to weakening the correlation between the leaf chlorophyll content and vegetation indices what then in practice may complicate chlorophyll retrieval from contact probe measurements from a leaf stack. It also implies that some chlorophyll-related indices unaffected by the contact probe measurement setup could be used in meta-studies even when combining various setups of the contact probe measurements at the leaf level. On the other hand, the effect of the contact probe measurement setup on chlorophyll indices combining wavelengths from both VIS and NIR (indices which are MS-affected) was present even after the z-score standardization, particularly in the beginning of the season. Therefore, we recommend taking into account this dependence on measurement setup, regardless the data standardization. Many MS-affected indices were better correlated with chlorophyll content in both species in comparison with MS-unaffected indices. Therefore, in terms of monitoring leaf chlorophyll content using the spectroscopic approach, these MS-affected indices should be used with the above-described caution.

Even if the vegetation indices are used for assessment of plant physiological status in various times of a vegetative season, it is still essential to take into consideration their possible changes induced by the particular contact probe measurement setup regarding the leaf stacking.

## Figures and Tables

**Table 1:** The first and the second columns list a name of an index and a formula of the index, respectively. The column R^2^-1L, 5L—shows the coefficient of determination for linear regression between the vegetation index and the chlorophyll content for a single leaf and a stack of leaves during the season. Underlined values of R^2^ correspond to a significant correlation. Indices defined as carotenoid- and water-related were excluded from the regression analysis (an empty lines). Months (May–October) show significance of the difference between indices computed from the reflectances of a single leaf and a leaf stack for each month separately tested by two-sample *t*-test, considered as significant if *p* < 0.05. According to the *p*-value, the level of significance is classified to three groups: not significant if *p* ≥ 0.05 coded in blue; significant if *p* = (0.05, 0.0000001) coded in red; very strongly significant if *p* < 0.0000001 coded in dark red. The majority of indices are listed in [5,22,24,31]

					*Populus tremula*					*Salix caprea*								
Index	Formula	R^2^		May	June	Jul	Aug	Sep	Oct	R^2^		May	June	Jul	Aug	Sep	Oct
		1L	5L							1L	5L						
BGI	=R450/R550	0.42	0.33							0.20	0.16							[25]
BI	=(R800+R670+R550)/sqrt(3)	0.01	0.10							0.00	0.00							[43]
Carter4	=R710/R760	0.52	0.42							0.40	0.33							[44]
CI	=(R800-R550)/R800	0.42	0.34							0.31	0.30							[43]
CRI550	=(1/R515)-(1/R550)																	[45]
CRI700	=(1/R515)-(1/R700)																	[45]
CTR	=R695/R760	0.40	0.29							0.36	0.22							[44]
CUR	=(R675·R690)/R683^3^	0.02	0.01							0.00	0.00							[46]
Datt	=(R850-R710)/(R850-R680)	0.51	0.44							0.35	0.35							[47]
Datt2	=R850/R710	0.50	0.41							0.37	0.38							[47]
DD	=(R749-R720)-(R701-R672)	0.53	0.42							0.39	0.32							[48]
DVI	=R800-R670	0.31	0.21							0.09	0.06							[49]
G	=R554/R677	0.21	0.15							0.12	0.10							[25]
Gitelson2	=(R750-R800/R695-R740)-1	0.39	0.24							0.41	0.31							[45]
GM94a	=R750/R700	0.47	0.32							0.43	0.35							[50]
gNDVI_780_	=(R780-R550)/(R780+R550)	0.42	0.34							0.32	0.31							[51]
GRg	=(R800/R550)-1	0.40	0.29							0.30	0.34							[45]
Macc01	=(R780-R710)/(R780-R680)	0.52	0.45							0.38	0.34							[52]
MCARI	=((R700-R670)-0.2·(R700-R550))·(R700/R670)	0.43	0.33							0.30	0.27							[7]
MCARI1	=1.2·(2.5·(R800-R670)-1.3·(R800-R550))	0.02	0.09							0.00	0.01							[53]
MCARI2	=((R750-R705)-0.2·(R750-R550))·(R750/R705)	0.54	0.36							0.44	0.31							[54]
MCARI2/OSAV2		0.55	0.36							0.43	0.28							[54]
MCARI/OSAVI		0.46	0.35							0.31	0.28							[7]
McM94	=R700/R670	0.37	0.28							0.28	0.23							[55]
MND	=(R750-R445)/(R750+R705-2·R445)	0.53	0.42							0.42	0.34							[15]
mND_705_	=(R750-R705)/(R750+R705-2·R445)	0.53	0.42							0.42	0.34							[15]
MNDVI1	=(R755-R745)/(R755+R745)	0.43	0.44							0.27	0.30							[56]
MNDVI8	=(R755-R730)/(R755+R730)	0.52	0.46							0.36	0.35							[56]
MNDVIre	=(R750-R705)/(R750+R705-R445)	0.52	0.41							0.42	0.34							[15]
MSAVI	=0.5·(2·R800+1-sqrt((2·R800+1)^2^-8·(R800-R670)))	0.28	0.22							0.06	0.06							[57]
MSI	=R1600/R820																	[58]
MSR	=((R800-R670)-1)/sqrt((R800/R670)+1)	0.08	0.09							0.11	0.06							[59]
MTCI	=(R754-R709)/(R709-R681)	0.51	0.44							0.39	0.38							[60]
N705	=(R705-R675)/(R750-R670)	0.51	0.42							0.39	0.33							[1]
N715	=(R715-R675)/(R750-R670)	0.52	0.46							0.37	0.36							[1]
N725	=(R725-R675)/(R750-R670)	0.50	0.48							0.37	0.36							[1]
NDII2	=(R820-R1650)/(R820+R1650)																	[61]
NDVI1	=(R800-R670)/(R800+R670)	0.09	0.10							0.11	0.06							[20]
NDVI_800680_	=(R800-R680)/(R800+R680)	0.10	0.10							0.11	0.06							[62]
NDVIre	=(R750-R705)/(R750+R705)	0.51	0.39							0.42	0.33							[50]
NDWI	=(R858-R1240)/(R858+R1240)																	[63]
NDWI_2130_	=(R858-R2130)/(R858+R2130)																	[64]
NMDI	=(R860-(R1640-R2130))/(R860+(R1640-R2130))																	[65]
NPCI	=(R680-R430)/(R680+R430)	0.25	0.16							0.13	0.08							[66]
OSAVI	=(1+0.16)·(R800-R670)/(R800+R670+0.16)	0.25	0.17							0.13	0.08							[30]
OSAVI2	=(1+0.16)·(R750-R705)/(R750+R705+0.16)	0.52	0.39							0.42	0.32							[54]
PRI	=(R570-R530)/(R570+R530)	0.21	0.15							0.15	0.18							[67]
PRI·CI-H	=(R680-R500)/R750																	[68]
PRI·CI-Y	=(R570-R530)/(R570+R530)·(R760/R700-1)																	[69]
PRIm1	=(R515-R530)/(R515+R530)	0.25	0.22							0.00	0.00							[68]
PSNDb	=(R800-R635)/(R800+R635)	0.30	0.23							0.35	0.19							[24]
PSNDc	=(R800-R470)/(R800+R470)																	[24]
PSSRa	=R800/R680	0.08	0.05							0.13	0.05							[24]
PSSRb	=R800/R635	0.27	0.16							0.40	0.24							[24]
PSSRc	=R800/R470																	[24]
RDVI	=(R800-R670)/(sqrt(R800+R670))	0.18	0.11							0.14	0.07							[70]
REIP	=(700+40·((Rre-R700)/(R740-R700)))/100	0.51	0.43							0.34	0.27							[71]
REP	=700+40·((((R670+R780)/2)-R700)/(R740-R700))	0.51	0.42							0.34	0.27							[72]
REP-Li	=700+40·((R670+R780/2)/(R740-R700))	0.51	0.42							0.34	0.27							[71]
RMSR	=((R750/R705)-1)/sqrt((R750/R705)+1)	0.50	0.38							0.42	0.35							[54]
RNIRCRI_550_	=((1/R515-1/R550)·R770)																	[45]
RNIRCRI_700_	=((1/R515-1/R700)·R770)																	[45]
Rre	=(R670+R780)/2	0.25	0.22							0.05	0.05							[71]
SIPI	=(R800-R445)/(R800-R680)	0.32	0.23							0.17	0.09							[73]
SIPI_680_	=(R800-R455)/(R800-R680)	0.32	0.24							0.12	0.08							[73]
SIPI_705_	=(R800-R455)/(R800-R705)	0.50	0.41							0.35	0.26							[73]
SR	=R800/R670	0.07	0.06							0.13	0.06							[49]
SR2	=R750/R710	0.51	0.41							0.41	0.37							[74]
SR3	=R750/R550	0.40	0.29							0.33	0.34							[75]
SRPI	=R430/R680	0.25	0.15							0.12	0.08							[73]
SRWI	=R858/R1240																	[76]
TCARI	=3·((R700-R670)-0.2·(R700-R550)·(R700/R670))	0.38	0.30							0.22	0.22							[77]
TCARI/OSAVI		0.42	0.33							0.27	0.25							[77]
TCARI2	=3·((R750-R705)-0.2·(R750-R550)·(R750/R705))	0.12	0.29							0.00	0.26							[54]
TCARI2/OSAVI2		0.41	0.35							0.28	0.35							[54]
TVI	=0.5·(120·(R750-R550)-200·(R670-R550))	0.08	0.09							0.00	0.01							[78]
VI_[700]_	=(R700-R670)/(R700+R670)	0.38	0.30							0.28	0.21							[79]
Vogelmann	=R740/R720	0.51	0.44							0.40	0.37							[80]
Vogelmann2	=(R734-R747)/(R715+R726)	0.52	0.45							0.38	0.36							[80]
WI	=R900/R970																	[81]

**Table 2:** Descriptive statistics of the reflectance difference (∆R_5L–1L_) between the reflectances measured on a leaf stack (5L) and a single leaf (1L) in % within VIS (400–700 nm), NIR (740–1140 nm), SWIR1 (1500–1800 nm) and SWIR2 (2000–2400 nm) regions for all data averaged from six months for *P. tremula* and *S. caprea*.

***P. tremula***	**VIS**	**NIR**	**SWIR1**	**SWIR2**
Variance	0.0%	0.2%	0.1%	0.0%
Max	3.2%	43.3%	14.0%	4.8%
Min	−3.9%	10.0%	0.7%	−0.7%
Mean	0.0%	28.7%	7.2%	1.3%
SD	0.6%	4.2%	2.4%	1.1%
***S. caprea***	**VIS**	**NIR**	**SWIR1**	**SWIR2**
Variance	0.0%	0.2%	0.1%	0.0%
Max	10.1%	37.4%	14.0%	6.0%
Min	−3.0%	6.3%	1.1%	−0.9%
Mean	0.1%	26.3%	7.2%	1.6%
SD	0.9%	5.0%	2.4%	1.2%
